# Sevoflurane induces cognitive impairment in young mice via autophagy

**DOI:** 10.1371/journal.pone.0216372

**Published:** 2019-05-20

**Authors:** Xiaoning Wang, Yuanlin Dong, Yiying Zhang, Tianzuo Li, Zhongcong Xie

**Affiliations:** 1 Department of Anesthesiology, Beijing Shijitan Hospital, Capital Medical University, Beijing, China; 2 Department of Anesthesia, Critical Care and Pain Medicine, Massachusetts General Hospital and Harvard Medical School, Charlestown, MA, United States of America; University of Pennsylvania, UNITED STATES

## Abstract

**Background:**

Anesthesia may induce neurotoxicity and neurocognitive impairment in young mice. However, the underlying mechanism remains largely to be determined. Meanwhile, autophagy is involved in brain development and contributes to neurodegenerative diseases. We, therefore, set out to determine the effects of sevoflurane on autophagy in the hippocampus of young mice and on cognitive function in the mice.

**Methods:**

Six day-old mice received 3% sevoflurane, for two hours daily, on postnatal days (P) 6, 7 and 8. We then decapitated the mice and harvested the hippocampus of the young mice at P8. The level of LC3, the ratio of LC3-II to LC3-I, and SQSTM1/p62 level associated with the autophagy in the hippocampus of the mice were assessed by using Western blotting. We used different groups of mice for behavioral testing via the Morris Water Maze from P31 to P37.

**Results:**

The anesthetic sevoflurane increased the level of LC3-II and ratio of LC3-II/LC3-I, decreased the p62 level in the hippocampus of the young mice, and induced cognitive impairment in the mice. 3-Methyladenine, the inhibitor of autophagy, attenuated the activation of autophagy and ameliorated the cognitive impairment induced by sevoflurane in the young mice.

**Conclusion:**

These data showed that sevoflurane anesthesia might induce cognitive impairment in the young mice via activation of autophagy in the hippocampus of the young mice. These findings from the proof of concept studies have established a system and suggest the role of autophagy in anesthesia neurotoxicity and cognitive impairment in the young mice, pending further investigation.

## Introduction

There are about 6 million children who receive various surgeries under anesthesia every year in the United States of America [1]. Sevoflurane, which is characterized by a low pungency, nonirritating odor, and a low blood/gas partition coefficient, is the most commonly used inhalation anesthetic among children [2]. However, increasing evidence has suggested that sevoflurane may induce neurotoxicity and neurocognitive impairment in young animals [3–7].

Specifically, sevoflurane has been shown to induce cellular apoptosis [8–13], endoplasmic reticulum stress [9, 14–16], synaptogenesis impairment [17–20], mitochondrial dysfunction [18, 20] and neuroinflammation [9, 21] *in vitro* and *in vivo* in mice. These effects may then lead to cognitive impairment in young mice [22]. However, the underlying mechanism by which sevoflurane induces the neurocognitive impairment in young mice remains mostly unknown.

Meanwhile, autophagy includes degradation of the intracellular material within the lysosome and recycling of the macromolecular constituents [23], which starts with the formation of the phagophore and the initial sequestering compartment that later expands into an autophagosome. Autophagic flux, the difference between the formation of the autophagosome and clearance of cargo by lysosomes [23], is associated with the development of neurodegenerative diseases [24–26]. Specifically, the illustration of the mechanisms responsible for impairing autophagic flux represents an essential area of research in the field of neurodegenerative diseases [27].

Evidence indicates that autophagy could be involved in anesthesia neurotoxicity [28]. Anesthesia that contains 2% sevoflurane and that is administered 5 hours daily for one day has been shown to activate autophagy in the 20 month-old male Sprague-Dawley rats [28]. Specifically, the anesthetic sevoflurane induced cognitive impairment and the increase in the levels of LC3-II (microtubule-associated protein one light chain 3-II) and the ratio of LC3-II/LC3-I in the hippocampus of the older rats as compared to those in the control group at 24 hours post-anesthesia. Moreover, it has been demonstrated in an *in vitro* study that propofol (200μM) or isoflurane (2.4% isoflurane for 24 hours) impaired autophagy flux and caused cell death [29]. However, the role of autophagy in the neurotoxicity of the brain in young mice remains unknown.

Autophagy includes initiation, elongation, maturation, and degradation [30]. During the process of elongation, LC3 is converted from the soluble form (LC3-I) to the autophagosome-associated form (LC3-II) during the process of elongation [31,32]. Thus, LC3 is the most widely monitored autophagy-related protein and is commonly used as a marker of autophagy [33,34]. Sequestosome-1(SQSTM1/p62) links aggregated proteins sequestered in the autophagosomes and is degraded in the autolysosomes [23]. It is one of the most studied autophagy receptor proteins [27] and is commonly regarded as both an autophagy substrate protein [23] and a cargo receptor for autophagic degradation of polyubiquitinated proteins [35, 36]. The reduction in p62 by degradation in autolysosomes has been suggested as an index of activation of autophagy [23].

Thus, the objective of the current proof of concept study is to assess the effects of sevoflurane on autophagy in the brain tissues of young mice. The hypothesis was that the anesthetic sevoflurane induces activation of autophagy in the hippocampus of young mice, leading to cognitive impairment in these young mice. In the current studies, we also used 3-methyladenine (3-MA), which blocks autophagic vesicle formation via inhibition of phosphoinositide 3-kinase, as an autophagy inhibitor [37].

## Materials and methods

### Mice anesthesia and treatment

The animal protocol was approved by the Standing Committee on Animals at Massachusetts General Hospital (Protocol number: 2006N000219, Boston, Massachusetts). We performed the experiments following the National Institutes of Health (NIH) guidelines and regulations. Efforts were made to minimize the number of animals used in the studies. For these studies, we used both male and female WT mice (C57BL/6J; Jackson Lab, Bar Harbor, ME), which we randomly assigned into either the control group or the sevoflurane anesthesia group. We did not assess potential sex differences in the current studies because we wanted first to establish a system to determine the effects of anesthesia on the autophagy in the young mice. Here, the mice received the sevoflurane during postnatal days (P) 6, P7 and P8, similar to what they received in our previous studies [18, 38–40], and then were decapitated at P8 for the hippocampus harvest at the end of the experiment. We used different groups of mice for the behavioral testing from P31 to P37 following administration of the anesthetic sevoflurane at P6, P7, and P8. The mice received the anesthetic sevoflurane (3%) plus 60% oxygen (balanced with nitrogen) as performed in our previous studies [38, 41]. The size of the induction chamber in the current study was 20 × 20 × 7 cm. The induction flow rate was 2 L/min for the first 3 minutes (for the induction of anesthesia) and then 1 L/min (for the maintenance of anesthesia). Mice in the control groups received 60% oxygen at an identical flow rate in similar chambers and were separated from their mothers as well. We monitored the anesthetic and oxygen concentrations continuously with a gas analyzer (Ohmeda; GE Healthcare, Tewksbury, MA). The temperature of the anesthetizing chamber was controlled by the DC Temperature Control System (FHC, Bowdoinham, ME), which serves as a feedback-based system for monitoring and controlling temperature, to maintain the rectal temperature of the mice as 37° ± 0.5°C. A previous finding [5] showed that anesthesia containing 3% sevoflurane administered for 2 hours did not significantly change the values of pH, partial pressure of oxygen, or partial pressure of carbon dioxide as compared with similar measurements in the control group. We, therefore, did not measure the blood gas of the mice in the current studies. For the intervention studies, we administrated 3-methyladenine (3-MA) at 30mg/kg [37], which was dissolved in normal saline with the concentration of 1 ug ul^-1^, (Sigma-Aldrich, USA) [37], through intraperitoneal injections for 60 minutes before each of the sevoflurane administrations. The mice in the control group received normal saline (NS, 30 ml/kg) via the intraperitoneal injection. Altogether, there was less than a 1% mortality rate among the mice in the studies. All the experiments were performed blindly.

### Morris water maze

We performed the Morris Water Maze (MWM) experiments using published protocols [38]. The P31 mice were tested using the MWM through four trials per day for 7 (P31 to P37) days. A video recording device was used to track the mice swimming in the pool. Escape latency (time to reach the platform) from P31 to P37 and platform crossing times (times the mice moved across the original area of the removed platform) for P37 were recorded in attempts to assess spatial learning and memory function. Mice body temperature was maintained by using active heating as described in the previous study [42]. Precisely, after every trial, each mouse was placed in a holding cage under a heat lamp for 5 minutes to dry out before returning to its regular cage.

### Harvest of mouse hippocampus and quantification of protein

We used different groups of mice for biochemistry studies. The mice were killed via decapitation at P8 immediately after the anesthesia administration when the mice were still completely anesthetized. The hippocampus of each of the mice was harvested and subjected to Western blotting analysis. We homogenized the harvested hippocampus on ice using an immunoprecipitation buffer (Tris-HCl: 10 mM, pH 7.4; NaCl: 150 mM; EDTA: 2 mM, Nonidet P-40: 0.5%) plus protease inhibitors (aprotinin: 1 μg/ml; leupeptin: 1 μg/ml, pepstatin A: 1 μg/ml). The lysates were then collected and centrifuged at 4°C, 13,000 rpm for 15 minutes. We then used a bicinchoninic acid protein assay kit (Pierce, Iselin, NJ, USA) to quantify the amount of protein as performed in the other studies [43].

### Western blotting analysis

We used the LC3B monoclonal antibody (Cell Signaling, Danvers, MA, USA; #3868) at 1:1000 dilution, SQSTM1/p62 monoclonal antibody (Cell Signaling, Danvers, MA, USA; #5114) at 1:1000 dilution, and a beta-Actin antibody (Sigma, St Louis, MO, USA; #A5441) at 1:5000 dilution for the quantitative Western blotting analysis as described by Xie et al. [44]. We analyzed the signal intensity using the Quantity One image analysis program (Bio-Rad, Hercules, CA, USA). We used beta-Actin to standardize the amounts of protein (e.g., calculating the ratio of the amount of LC3-II to the amount of beta-Actin) and to limit disparities in the quantity of protein loaded. We expressed the protein levels as a percentage when comparing them to those in the control condition.

### Statistics

Data regarding biochemistry changes were expressed as mean ± standard deviation (SD). Data regarding changes in escape latency were expressed as mean ± standard error of the mean (SEM). The data for platform crossing time was not distributed normally and thus were expressed using the median and interquartile range. The number of samples was 10–11 per group in the behavioral studies and 6–8 per group in the biochemistry studies. These numbers were selected according to the results obtained in our previous studies [38, 39]. A two-way ANOVA with repeated measurements was used to evaluate the difference in escape latency through the MWM by comparing mice in the anesthesia group with those in the control group. Post hoc analysis was used to contrast the change in escape latency for each day during the MWM test, and the cut-off α was Bonferroni adjusted. The Mann-Whitney U-test was used to compare the platform-crossing times between the mice in the anesthesia group and the mice in the control group. Finally, a two-way ANOVA was used to evaluate the interaction between the group (control vs. anesthesia) and treatment (3-MA vs. normal saline) on the amounts of LC3-II, SQSTM1/p62, and the LC3-II to LC3-I ratio. A two-tailed hypothesis testing was used. Statistical significance was defined as P<0.05. We used Prism 6 (GraphPad, La Jolla, CA, USA) to evaluate all of the data.

## Results

### Sevoflurane anesthesia increased the LC3-II level and the ratio of LC3-II to LC3-I in the hippocampus of young mice

Anesthesia containing 3% sevoflurane administered for 2 hours daily for three days (multiple exposures of sevoflurane) in P6 mice has been reported to induce cognitive impairment in the mice [38]. We, therefore, set up an experiment to determine the effects of the sevoflurane anesthesia on autophagy in the hippocampus of the young mice.

**Fig 1:**
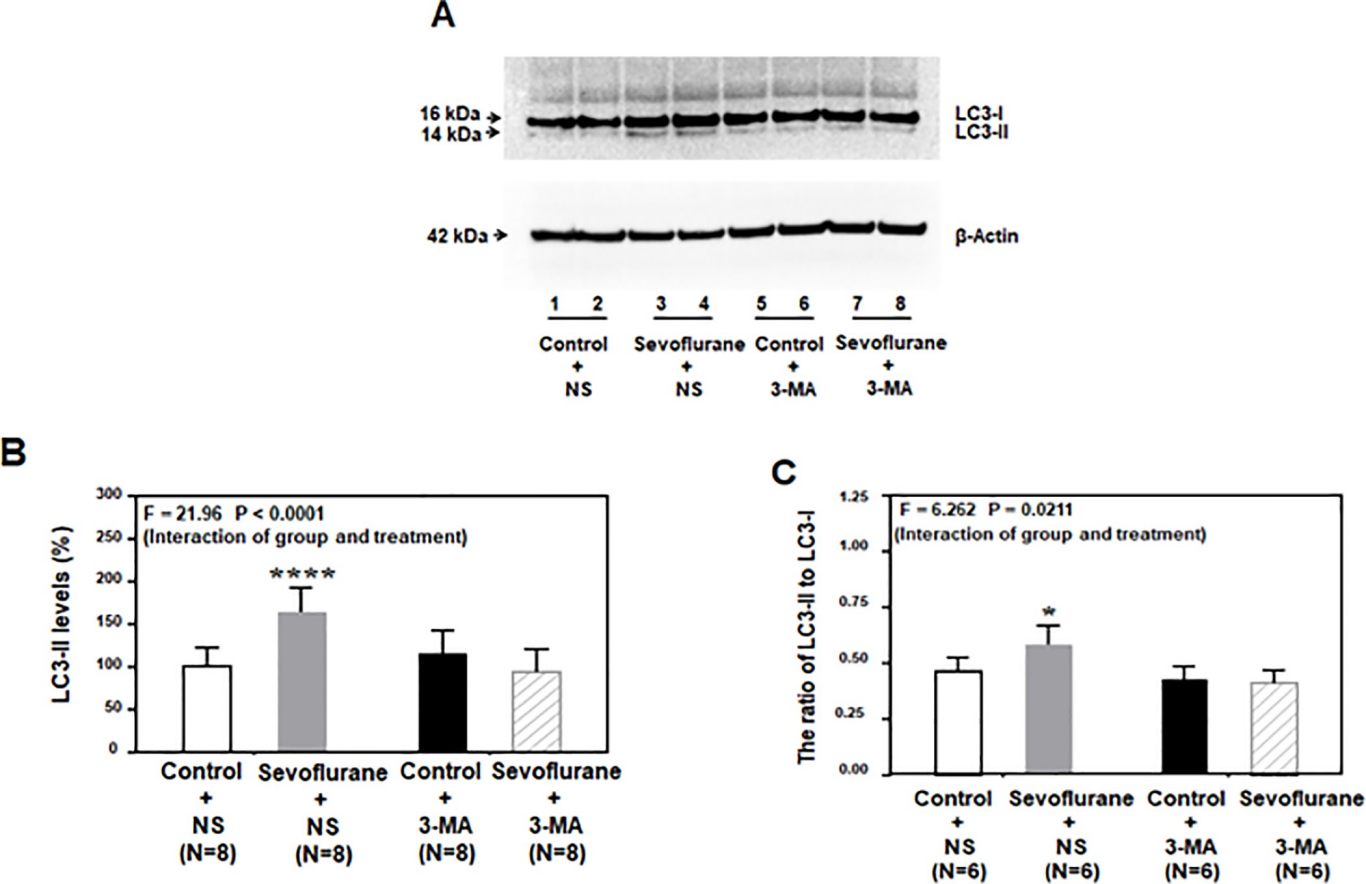
Sevoflurane increased LC3-II levels in the hippocampus of young mice. **A**. Anesthesia containing 3% sevoflurane administered for 2 hours daily for three days in P6 mice increased their levels of LC3-II (lanes 3 to 4) compared to those in the control condition (lanes 1 to 2). 3-MA treatment alone (lanes 5 to 6) did not alter the levels of LC3-II as compared to those in the control condition, but the 3-MA treatment (lanes 7 to 8) attenuated the sevoflurane-induced increase in the LC3-II levels of mice compared those receiving the anesthetic sevoflurane (lanes 3 to 4). There was no significant difference in the beta-Actin levels among these treatments. **B**. Quantification of the Western blot showed that the anesthetic sevoflurane increased the LC3-II levels (gray bar versus white bar) and that 3-MA attenuated the sevoflurane-induced increase in the LC3-II levels (net bar versus gray bar) (F = 21.96, P < 0.0001, two-way ANOVA). **C**. The quantification of the Western blots showed that the anesthetic sevoflurane increased the ratio of LC3-II/LC3-I in mice compared to those in the control condition (white bar versus black bar), and 3-MA attenuated the sevoflurane-induced increase in the ratio of LC3-II/LC3-I (net bar versus gray bar) (F = 6.262, P = 0.0211, two-way ANOVA). LC = microtubule-associated protein 1 light chain; 3-MA = 3-Methyladenine; ANOVA = analysis of variance; NS = normal saline.

The immunoblotting of both LC3-II and LC3-I showed that the anesthetic sevoflurane (Fig 1A; lane 3–4) increased the levels of LC3-II and the ratio of LC3-II/LC3-I in the hippocampus of mice compared those in the control condition (Fig 1A; lane 1–2). Treatment of 3-MA alone (Fig 1A; lane 5–6) did not significantly alter the levels of LC3-II and the ratio of LC3-II/LC3-I compared to those in the control condition (Fig 1A; lane 1–2). The 3-MA treatment (Fig 1A; lane 7–8) attenuated the sevoflurane-induced increase in the levels of LC3-II and the ratio of LC3-II/LC3-I in the hippocampus of the young mice compared to those in the saline treatment group.

The quantification of the Western blot showed that the anesthetic sevoflurane increased the level of LC3-II (Fig 1B, gray bar; P = 0.0002, N = 8, two-way ANOVA with Bonferroni multiple comparisons test) and the ratio of LC3-II/LC3-I (Fig 1C, gray bar; P = 0.0230, N = 6, two-way ANOVA with Bonferroni multiple comparisons test) in the hippocampus of mice compared to those in the control condition (Fig 1B and 1C; white bar).

Next, a two-way ANOVA showed that there was a significant interaction between the group (control versus sevoflurane) and treatment (saline versus 3-MA) in regards to the level of LC3-II (Fig 1B; F = 21.96, P < 0.0001, two-way ANOVA) and LC3-II/LC3-I ratio (Fig 1C; F = 6.262, P = 0.0211, two-way ANOVA). These data showed that sevoflurane was able to induce the activation of autophagy and that 3-MA attenuated such activations in the hippocampus of the young mice.

### Sevoflurane anesthesia in young mice decreased the p62 level in the hippocampus of young mice

**Fig 2:**
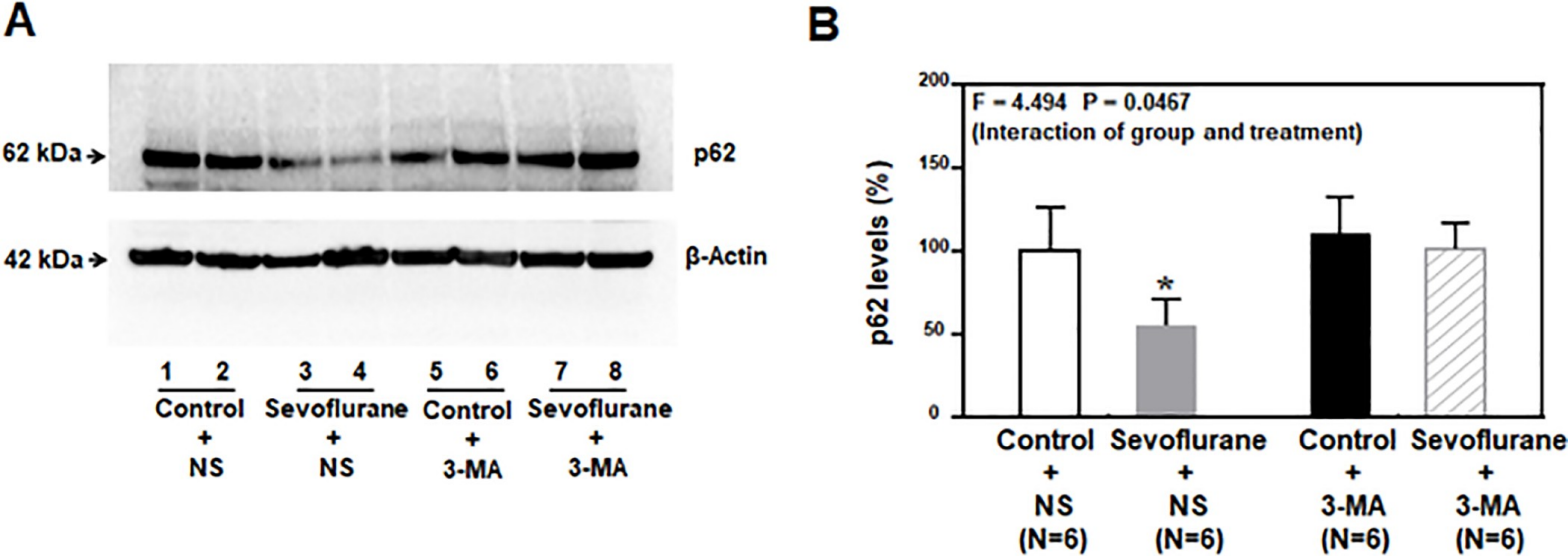
Sevoflurane decreased p62 level in the hippocampus of young mice. **A**. The anesthetic sevoflurane decreased the levels of p62 (lanes 3 to 4) in mice compared to those in the control condition (lanes 1 to 2). The 3-MA treatment alone (lanes 5 to 6) did not significantly alter the p62 levels in mice to those receiving saline treatment, whereas the pretreatment of 3-MA (lanes 7 to 8) attenuated the sevoflurane-induced decrease in the p62 levels. There was no significant difference in the beta-Actin levels among these treatments. **B**. The quantification of the Western blot showed that the anesthetic sevoflurane attenuated the p62 levels (gray bar versus white bar), and 3-MA attenuated the sevoflurane-induced reduction in the levels of p62 (net bar versus gray bar) (F = 4.494, P < 0.0467, two-way ANOVA). p62 = Sequestosome 1(SQSTM1); 3-MA = 3-Methyladenine; ANOVA = analysis of variance.

The immunoblotting of p62 showed that the anesthetic sevoflurane (Fig 2A; lanes 3 and 4) decreased the level of p62 in the hippocampus of the young mice compared to those in the control condition (Fig 2A; lanes 1 and 2). The 3-MA treatment alone (Fig 2A; lanes 5 and 6) did not significantly alter the p62 level compared to those in the saline condition (Fig 2A; lanes 1 and 2) whereas the 3-MA treatment (Fig 2A; lanes 7 and 8) attenuated the sevoflurane-induced decrease in the p62 level in the hippocampus of the young mice.

The quantification of the Western blot showed that the anesthetic sevoflurane decreased the p62 level (Fig 2B, gray column; P = 0.0061, N = 6, two-way ANOVA with Bonferroni multiple comparisons test) in the hippocampus of mice (harvested at P8) as compared to those in the control condition (Fig 2B; white column). A two-way ANOVA showed that there was a significant interaction between the group (control vs. sevoflurane) and treatment (saline and 3-MA) in regards to the levels of p62 (Fig 2B; F = 4.494, P = 0.0467). These data indicated that the over-activated autophagy induced by multiple sevoflurane exposures might be one of the underlying mechanisms of sevoflurane-induced neurotoxicity in the developing brain and that 3-MA, the autophagy inhibitor, could attenuate the sevoflurane-induced activation of autophagy.

### 3-MA ameliorated the sevoflurane-induced cognitive impairment in young mice

Our previous studies demonstrated that multiple exposures to sevoflurane induced cognitive impairment in young mice [38, 39]. Given the findings that 3-MA, the inhibitor of autophagy, was able to attenuate the sevoflurane-induced activation of autophagy, we then asked whether or not 3-MA could attenuate the sevoflurane-induced cognitive impairment in the young mice subjected to the MWM experiments to test whether or not activation of autophagy contributed to the sevoflurane-induced cognitive impairment in these young mice.

**Fig 3:**
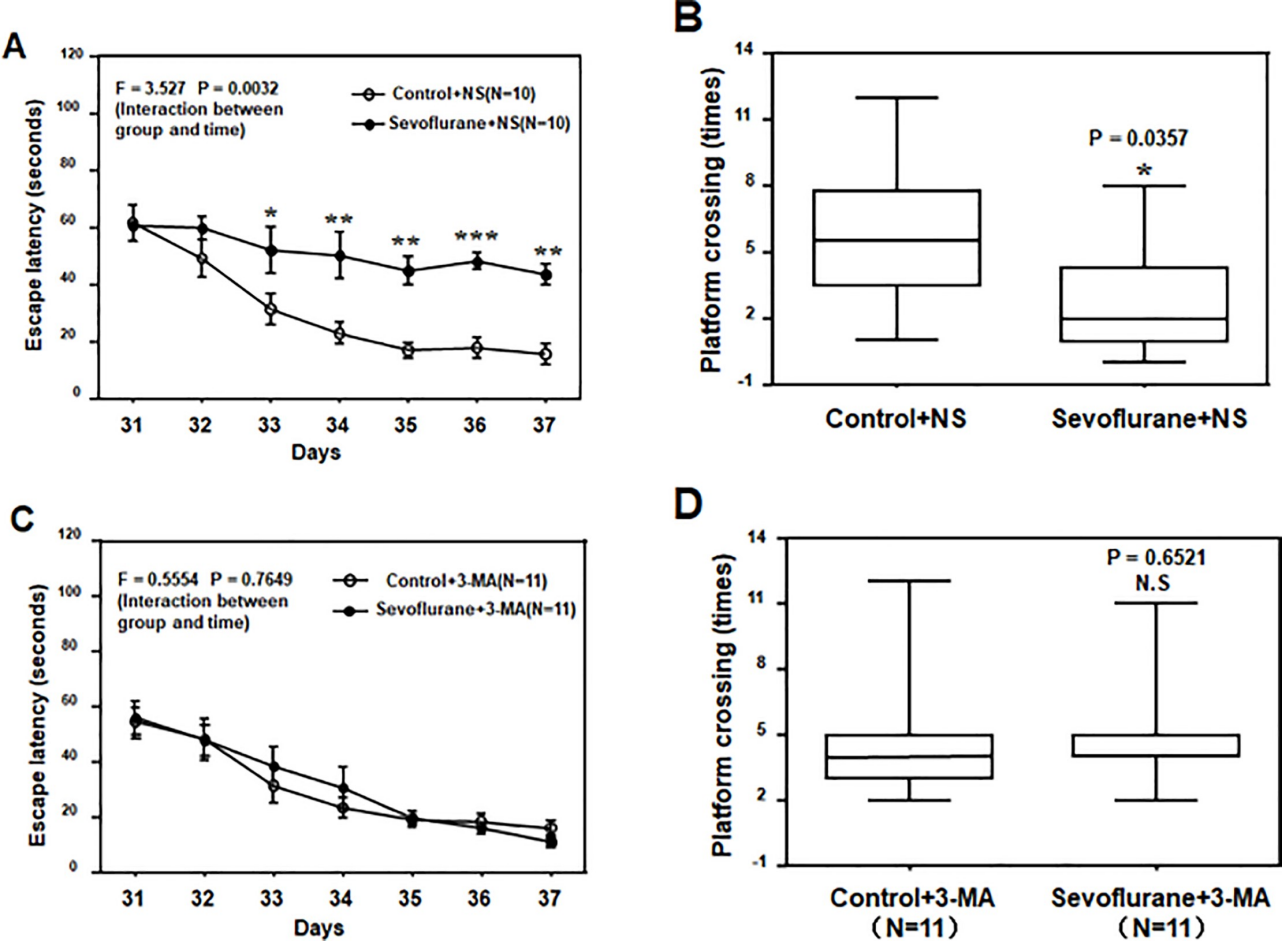
3-MA mitigated the sevoflurane-induced cognitive impairment in young mice. **A**. The two-way ANOVA with repeated measurement analysis showed that there was statistically significant interaction between treatment and group based on escape latency of the Morris Water Maze (MWM) between the control plus saline-treated (N = 10 in each group) and sevoflurane (3% for 2 h daily for 3 days) plus saline-treated (N = 10 in each group) mice (F = 3.527, P = 0.0032). The post hoc (Bonferroni) test showed that the mice given the sevoflurane plus saline treatment had longer escape latencies compared to mice given the control plus saline treatment at day P33 to P37. **B**. The Mann-Whitney test showed that the mice given the sevoflurane plus saline treatment (N = 10 in each group) had less platform crossing times compared with mice given the control plus saline treatment (N = 10 in each group) in the MWM test. **C**. The two-way ANOVA with repeated measurement analysis showed that there was no statistically significant interaction between treatment and group based on the escape latency of MWM between the control plus 3-MA-treated (N = 11 in each group) and sevoflurane plus 3-MA-treated (N = 11 in each group) mice. **D**. The Mann–Whitney test showed that there was no significant difference in platform crossing times of mice in the MWM between the control plus 3-MA treatments (N = 11 in each group) and sevoflurane plus 3-MA treatments (N = 11 in each group). 3-MA = 3-Methyladenine; ANOVA = analysis of variance; NS = normal saline.

Among the mice that received pretreatment via normal saline, the anesthetic sevoflurane increased their escape latency in the MWM (tested from P31 to P37) (Fig 3A; F = 3.527, P = 0.0032, Two-Way ANOVA) and reduced their platform crossing times (tested at P37) (Fig 3B; P = 0.0357, Mann-Whitney test) compared to those in the control condition. In contrast, among mice that received the pretreatment of 3-MA, the anesthetic sevoflurane did not increase their escape latency in the MWM from P31 to P37 (Fig 3C; F = 0.5554, P = 0.7649) or the platform crossing times at P37 compared to those in the control condition (Fig 3D; P = 0.6521, Mann-Whitney test). These data demonstrated that sevoflurane could induce neurotoxicity and cognitive impairment through activation via autophagy, which might be attenuated by 3-MA, the inhibitor of autophagy.

## Discussion

The role of autophagy in regards to anesthesia neurotoxicity in young mice has not been primarily investigated. In the current proof of concept study, we established a system to assess the activation of autophagy *in vivo* and demonstrated that the anesthetic sevoflurane induced activation of autophagy and that the inhibition of autophagy by 3-MA was able to attenuate the sevoflurane-induced cognitive impairment in the young mice. These data suggest the potential role of autophagy in regards to anesthesia neurotoxicity in young mice.

Although many studies have determined the potential mechanisms of anesthesia neurotoxicity in young mice, the exact mechanisms remain largely to be determined. Thus, future studies seeking to reveal such mechanisms are of great importance in helping to improve anesthesia safety and to provide better postoperative outcomes for children.

Autophagy is known to be triggered as a cellular reaction in response to physiological changes such as nutrient depletion and starvation [45]. Moreover, recent studies have also demonstrated that autophagy is closely associated with neurotoxicity [46, 47]. However, the effects of sevoflurane, the most commonly used anesthetic in children, on autophagy remains mostly unknown, and the precise mechanisms are underlying sevoflurane neurotoxicity still needs to be further explored.

In the present study, we first found that the degree of autophagy, measured by the expression of LC3-II and the ratio of LC3-II/LC3-I, was significantly increased in the hippocampus of young mice after administration of the anesthetic sevoflurane (Fig 1). The anesthetic sevoflurane decreased the levels of p62 in the hippocampus of young mice (Fig 2). The changes in the levels of LC3-II, the LC3-II/LC3-I ratio, and p62 levels induced by sevoflurane were mitigated by the treatment of 3-MA, the inhibitor of autophagy. Taken together, these data suggest that the anesthetic sevoflurane can induce activation of autophagy in the brain tissues of young mice.

Moreover, the sevoflurane-induced cognitive impairment in the young mice was also mitigated by the treatment of 3-MA. These results indicate that activation of autophagy contributes, at least partially, to the neurotoxicity and neurobehavioral deficits induced by the anesthetic sevoflurane.

In previous studies, the exact role of autophagy on neurotoxicity has remained controversial [48, 49]. The mechanism by which autophagy contributes to the neurotoxicity remains largely to be determined. Therefore, future research should investigate the dose- and time-dependent effects of anesthesia on autophagy *in vitro* and *in vivo*. The outcomes from the current studies have only established a system and generated a hypothesis regarding the role of autophagy in anesthesia neurotoxicity, which should help promote further investigation into anesthesia neurotoxicity in young brains.

Autophagy plays an important physiological role in the clearance of degraded proteins. However, excessive autophagy may also cause autophagic cell death, and impairment of autophagy flux may promote cell death by apoptosis [50, 51]. The findings in the current studies that sevoflurane induced the elevation in LC3II, decrease in P63 and 3-MA-regulated inhibition of P62 demonstrated that the sevoflurane-induced excessive autophagy likely led to the cognitive impairment in the young mice. However, the current findings could not rule out the possibility that cognitive impairment resulted from the sevoflurane-induced defected autophagy and cellular apoptosis. The future studies to determine the effects of sevoflurane on the balance of the autophagy process are warrened.

A recent study by Cheng et al. showed that oxygen-glucose deprivation enhanced the accumulation of autophagosome through the increases in the formation of autophagosomes and decreases in the autophagosome clearance [52] in SY5Y cells. Moreover, postconditioning with sevoflurane mitigated the oxygen-glucose deprivation-induced autophagosome accumulation, leading to cellular protection in the cells [52]. However, the treatment of sevoflurane in the studies by Cheng et al. was 1%, 2%, 3%, 4% and 5% for 15 minutes [52]. The anesthesia used in the current studies was 3% sevoflurane 2 hours daily for three days in young mice at P6, P7, and P8. We postulate that different concentrations of sevoflurane with different durations will have different effects on autophagy. Future studies will test this hypothesis in both cellular levels and animals.

Overall, this study has several limitations. First, Western blotting, which helped reveal the conversion of LC3-I to LC3-II and detect p62 levels, was the only method used to demonstrate autophagy in the hippocampus of young mice after the administration of the anesthetic sevoflurane. Electron microscopy of autophagosomes or green fluorescent protein-LC3 emergence, which occurs after plasmid transfection by immunofluorescence microscopy, is often used in combination with immunoblot assays to detect autophagy [53]. However, Western blot assays have also been considered as a reliable method to check for autophagy in many studies [54,55]. Second, only pharmacologic inhibition has been adopted to examine the role of autophagy as genetically engineered animals defective in the autophagy process were not available in our present experimental conditions. In future studies, we may need to develop more sophisticated methods to detect the effects of anesthesia on autophagy levels in the hippocampus of young mice. Finally, the increased levels of LC3-II and the ratio of LC3-II to LC3-I could also be the result of impaired autophagic flux as the decreased clearance of LC3-II. The future studies will systematically determine the effects of sevoflurane on the metabolism of LC3-II, including both generation and clearance.

In conclusion, we discovered the activation of autophagy in the hippocampus after the sevoflurane anesthesia in the young mice, and that inhibition of autophagy with 3-MA ameliorated the cognitive impairment in the young mice. These results from the proof of concept study have established a system and currently suggest the potential role of autophagy in anesthesia neurotoxicity in young mice, which could promote more research into anesthesia neurotoxicity in the future.

## Supporting information

S1 Data The compressed file contains the raw data of the figures in the manuscript, the corresponding statistical processing and the generated graphs by the software of GraphPad Prism. (RAR)

S2 Data In the compressed file, the bands of LC3-I, LC3-II, p62 and the corresponding internal reference protein, beta-Actin, are included. (RAR)
